# Risky Sexual Behavior of Young Adults in Hong Kong: An Exploratory Study of Psychosocial Risk Factors

**DOI:** 10.3389/fpsyg.2021.658179

**Published:** 2021-03-22

**Authors:** Heng Choon (Oliver) Chan

**Affiliations:** Teaching Laboratory for Forensics and Criminology, Department of Social and Behavioral Sciences, City University of Hong Kong, Hong Kong, China

**Keywords:** sexual risk taking, risky sexual behavior, sexual behavior, psychosocial risk factors, young adults

## Abstract

There is limited knowledge of the prevalence and nature of risky sexual behavior (RSB) among young adults in Hong Kong. This cross-sectional study explored the psychosocial risk factors of RSB with a sample of 1,171 Hong Kong university students (aged 18–40 years). Grounded in the theoretical propositions of several criminological theories (i.e., the theories of self-control, general strain, social learning, social control, and routine activity), engagement in three types of RSB (i.e., general, penetrative, and non-penetrative) was studied alongside a range of psychosocial risk factors. Relative to female participants, male participants reported significantly higher mean levels of general, penetrative, and non-penetrative RSB. Male participants also reported significantly higher mean levels of negative temperament, use of alcohol and other drugs, and paraphilic interests than female participants, who reported significantly higher mean levels of self-control and social bonds than their male counterparts. The results of multivariate analyses (i.e., OLS regressions) revealed that, to a large extent, the male and female participants shared a similar set of psychosocial risk factors (i.e., use of alcohol and other drugs, and paraphilic interest) for their involvement in general, penetrative, and non-penetrative RSB. Furthermore, a high level of negative temperament was significantly associated with penetrative RSB for both genders, while a high level of perceived neighborhood disorganization was found to be an important factor in the participation of females in general, penetrative, and non-penetrative RSB. The findings of this study may have important implications for practice in regard to reducing, if not entirely preventing, the tendency to engage in RSB.

## Introduction

Risky sexual behavior (RSB), such as unprotected vaginal, oral, or anal intercourse, incorrect or inconsistent use of contraceptive measures, and sex with multiple partners and/or high-risk partners (i.e., intravenous drug users), has an enormous global impact. Around the world, over a million people are infected with a sexually transmitted infection (STI) each day (World Health Organization, 2019). A population-based geospatial household survey and test conducted with 881 participants (aged 18–49 years) in Hong Kong from 2014 to 2016 found that the prevalence of *Chlamydia trachomatis* (CT) was low overall (1.4%) but relatively high (5.8%) among sexually active young females (aged 18–26 years) (Wong et al., 2017). Chlamydia is a common bacterial infection caused by the CT bacteria that is transmitted through sexual contact with infected persons and is the most frequently notified STI in the European Union and the United States. In addition to STIs (including CT and HIV infections), RSB can lead to long-term poor reproductive health outcomes, such as infertility and pelvic inflammatory disease (Abajobir et al., 2017). The most significant risks for sexually active people are HIV infection, other STIs (e.g., chlamydia, gonorrhea, and syphilis), and unintended pregnancy (Hoyle et al., 2000). At a societal level, STIs can place a significant burden on a country's health care system.

Adolescents and young adults are at a heightened risk for negative sexual health outcomes in part due to their high rates of unprotected sex with multiple partners. The Global Burden of Disease Study that includes annual assessments for 188 countries from 1990 to 2013 found that unsafe sex practiced by young people aged 10–24 years was a risk factor for an increased level of disability-adjusted life-years (Mokdad et al., 2016). In the United States, for instance, <50% of sexually active adolescents reported using condoms on a regular basis (Martinez et al., 2011), and 14% of sexually active adolescents reported four or more lifetime sexual partners, which was higher than other age groups (DiClemente et al., 2010). Fetene and Mekonnen (2018) found in their sample of 524 young adults in Addis Ababa with a mean age of 21 years that 42% of the participants had more than one sexual partner and 47% of them did not use a condom during sexual intercourse in the past 12 months. In Chiang Mai, Thailand, Pinyopornpanish et al. (2017) found that most of their adolescent and young adult participants (35% of 1,744 participants) had not used a condom during sexual intercourse in the past 3 months (i.e., 66% of those aged 15–19 years, 80% of those aged 20–29 years, and 89% of those aged 30–39 years), and 41% of participants aged from 15 to 19 years reported having had more than one sexual partner in the past 3 months.

Understanding RSB remains pertinent not just from a public health standpoint but also in the criminal justice arena (because, for example, engaging in RSB may lead to perpetrating such criminal behavior as nonconsensual sexual intercourse). Importantly for this study, the high rates of RSB among young adults and the resulting high risk of negative sexual health outcomes highlight the need to understand the risk factors associated with RSBs so that an effective and timely identification of and intervention for those who are at the greatest risk for these outcomes can be made. More importantly, most of the literature on the topic is explored from a public health or clinical perspective. Hence, examining the psychosocial risk factors of RSB through a criminological lens will further advance our knowledge in this area and contribute to literature, particularly when the escalation of RSB to more serious deviant sexual behavior (e.g., sexual assault, rape) is possible.

## Theoretical Background

Self-control has been widely adopted to explain an individual's intention to engage in offending and risky behavior. The self-control theory (a.k.a. the general theory of crime) proffered by Gottfredson and Hirschi (1990) posits that individuals with less self-control are more likely to engage in risky and offending behavior in pursuit of immediate satisfaction without considering the potential consequences. Individuals who are low in self-control are likely to manifest six key characteristics: they are risk-seeking, impulsive, short-tempered, and self-centered, and they prefer physical over mental activities and simple over complex tasks (Muraven et al., 2006). The developmental phase of these individuals is often characterized by early exposure to criminogenic environments that did not allow them to develop sufficient control over their behavior. It is argued that the self-control personality trait, formed between the ages of 6 and 10, is relatively stable over the lifespan of individuals (Hirschi and Gottfredson, 1994), irrespective of their demographic characteristics, such as age, gender, culture, and social class (Vazsonyi and Klanjšek, 2008). Freeman and Muraven (2010) found that relative to those who were high in self-control, individuals who were low in self-control were more likely to take excessive risks. Low self-control individuals have been found by empirical studies to be more likely to engage in various types of risky behavior, such as RSB (Griffin et al., 2012; Kahn et al., 2015), reckless driving (Ferreira et al., 2009; Ellwanger and Pratt, 2014), the use of alcohol and other drugs (Vazsonyi et al., 2006; King et al., 2011), and general risky behavior and minor delinquency (Lu et al., 2013; Chan and Chui, 2017).

According to the general strain theory, the experiencing of stressors (strains) in individuals may interact with their individual characteristics to amplify the risk of engaging in maladaptive behaviors, such as risky behavior and criminal activities (Agnew, 2002). These manifestations of negative coping arise in response to adverse conditions, events, or treatment. Negative emotions (e.g., anger, depression, and frustration) function as a stimulus for action that triggers a progression from the experience of strain through to risky and offending behavior (Agnew, 1992). Exposure to strains may produce negative emotions that demand corrective action (Agnew et al., 2002). For instance, Leith and Baumeister (1996) found that individuals who experienced bad moods were more likely to engage in risk-taking behavior due to impaired self-regulation. Auerbach et al. (2007) noted that individuals who exhibited high levels of neuroticism and emotional regulation deficits were more likely than others to report increased engagement in risky behaviors following increases in the symptoms of negative emotions (e.g., depression, anxiety). A similar finding was reported in Auerbach et al. (2010) study of Chinese adolescents, in which individuals who exhibited high levels of neuroticism and a tendency to adopt maladaptive cognitive emotional regulation strategies were more likely than others to report greater engagement in risky behaviors following increases in the symptoms of depression. According to Stuewig and Tangney (2007), shame and guilt both reflect self-evaluative judgments that may result in negative and uncomfortable emotions (e.g., depression, anxiety, and anger), which may lead to maladaptive behavior, such as substance abuse and RSB. Later, Stuewig et al. (2015) reported that shame-prone children were more likely to engage in RSB (e.g., unprotected sex) and use illegal drugs in young adulthood.

The learning approach, which includes Sutherland's (1947) differential association theory and Akers's (1985) social learning theory, postulates that risky, delinquent, and offending behavior is learned through close social interaction with family and peers in the form of reinforcement or reward and punishment. Differential reinforcement or punishment simply refers to the net balance of expected social and/or nonsocial rewards and costs associated with different behavior (Akers, 1997). Social reinforcement involves “not just the direct reactions of others present while an act is performed, but also the whole range of tangible and intangible rewards valued in society and its subgroups” (Akers, 1997, p. 55), such as financial rewards, positive facial expressions, and verbal approval from significant others. Nonsocial reinforcements, conversely, are “unconditioned positive and negative effects of physiological and psychological stimuli” (Akers, 1998, p. 71), such as the psychophysiological effects of a stimulant. Bandura (1973) stressed that the imitation of observed behavior is also a primary process of behavioral learning. The probability of an individual learning the observed behavior increases when he/she anticipates incentives. Nonetheless, the impact of such exposure varies greatly according to the frequency, duration, intensity, and priority of different associations (Akers, 1998). It is suggested that adverse childhood and/or adolescence traumatic experiences (e.g., physical and/or sexual abuse and witnessing domestic violence) predisposed an individual to subsequent negative life outcomes (e.g., sexual deviant and criminal behavior; Chan et al., 2011; Chan, 2015, 2019a). Even though the primary social groups (e.g., family and peers) tend to have a strong influence on the behavioral learning process, secondary and reference groups (e.g., the school system, colleagues and work groups, the mass media, the Internet, and computer and mobile games) can be similarly important to normative definitions (e.g., attitudes, values, norms, and beliefs) in the learning process (Warr, 2002). For instance, Taylor et al. (2007) reported that gang members were more likely than non-gang members to engage in delinquent lifestyles, such as to be involved in unsupervised activities with peers, to hang out with peers where alcohol and/or other drugs were available, and to engage in a considerably greater amount of delinquent and risky behavior. A plethora of literature has consistently found support for a positive relationship between the use of alcohol and/or other drugs and involvement in risky behavior (e.g., unsafe sex, going on a blind date, hitchhiking; Windle, 1994; Zapolski et al., 2009; Roth et al., 2015). Specifically, Leigh (1990) reported a strong correlation between the frequency of using alcohol or other drugs in conjunction with sexual activity and the frequency of engaging in RSB in a sample of homosexual adults. Staton et al. (1999) found that the increased use of alcohol and marijuana at younger ages was significantly related to riskier sexual activity and increased use of alcohol and marijuana as young adults.

A positive relationship has also been noted between paraphilic interests and/or activities and RSB. Consistent with some theories of sexual offending, Lalumière et al. (2005) posited that an individual's paraphilic behavior may be influenced by his/her degree of impulsivity or propensity to engage in sexual and nonsexual risky behavior. Empirical support for the roles of risk-taking and impulsivity in the expression of paraphilic interests and activities has accumulated (e.g., Kafka, 1997, 2001; Långström and Seto, 2006; Marshall, 2007; Chan, 2021). Dawson et al. (2016) asserted that low levels of general inhibition, as a result of greater degrees of sensation-seeking and impulsivity, may lead to the development of atypical sexual interests. Arguably, a high sex drive and a strong preference for novelty precede or contribute to the development of paraphilic interests, such that some individuals may habituate to conventional sexual partners and activities (Kafka, 2001). Studies have found that individuals diagnosed with paraphilias report a high number of sexual partners, high rates of sexual behavior, and high sexual appetite and preoccupation (Kafka and Hennen, 2003; Långström and Hanson, 2006; Kafka, 2009). As such, the development of new (atypical) sexual interests may be related to an individual's impulsivity and risk-taking tendencies.

Hirschi's (1969) social control theory explains offending behavior in terms of the strength or weakness of social bonds. According to this theory, individuals with strong social bonds to conventional society (e.g., parents, prosocial peers, and school) are less likely to become involved in risky, delinquent, or criminal activities. There are four key elements of a social bond: (1) attachment (i.e., an individual's affective or emotional ties toward parents, peers, and school), (2) commitment (i.e., an individual's investment in prosocial behavior, including a willingness to do what is promised and a respect for the expectations of others over delivering on a promise), (3) involvement (i.e., an individual's active participation in prosocial activities, such as sports, religious practices, and community services), and (4) belief (i.e., an individual's respect for the moral validity of societal norms and regulations). These elements are strongly correlated, and their combined effect is likely to be stronger than their individual effects. Laundra et al. (2002) argued that attachment and commitment to prosocial individuals and activities are likely to increase an individual's morality belief, which may reduce their propensity to engage in delinquent or criminal activities. Empirical research has found support for a positive association between weak social bonds (e.g., parental and school attachment) and involvement in various types of risky behavior such as RSB (e.g., Peterson et al., 2010; Simons et al., 2016) and general risky and delinquent behavior (e.g., Chan and Chui, 2013, 2015). Specifically, a study of adolescent females conducted by Taylor-Seehafer and Rew (2000) found that positive family and school connectedness, the presence of caring adults, and the development of a healthy sexuality were significant protective factors against the early initiation of sexual activity, which may result in such RSB as the early initiation of sexual intercourse, unprotected sexual intercourse, and the inconsistent use of condoms and other forms of barrier contraception. A recent meta-analysis conducted by Kim and Miller (2020) reported that insecure attachment styles were related to RSB. The relationship between attachment anxiety and having multiple partners was stronger when the average age of the participants was higher and when the study population was specifically an at-risk population.

Situational mechanisms, such as routine activities and lifestyle choices, are also commonly used to explain risky and offending behavior. In their routine activity theory, Cohen and Felson (1979) hypothesized that the probability of a crime occurring is largely influenced by the convergence in time and space of three core elements in the daily routines of individuals: (1) a potential or motivated offender, (2) a suitable or attractive target, and (3) an absent or ineffective guardian who would be otherwise capable of protecting against a violation. The lack of any of these elements diminishes the possibility of a potential crime (Felson and Cohen, 1980). This theoretical model, which was originally developed to explain victimization as the outcome of legitimate and routine daily activities that expose poorly guarded targets to potential offenders in close proximity, has later been extended to describe risky, delinquent, and offending behavior. Simply put, an individual's tendency to engage in risky behavior is largely correlated with his/her specific daily activities (e.g., prosocial or deviant peer association), lifestyle (e.g., living environment), and status (e.g., personal characteristics). Osgood et al. (1996) asserted that individuals who spend substantial times in “unstructured socializing” with deviant peers and without adult supervision may be at an increased risk of engaging in risky and criminal activities. Indeed, studies have reported findings that an affiliation with deviant peers was associated with more RSB [e.g., STIs, unintended pregnancies; see Lansford et al. (2014)] and the use of alcohol and other drugs (Sanchagrin et al., 2017). Tangible and intangible rewards (e.g., status or reputation within a group) may also encourage initial and persistent participation in risky and criminal activities [e.g., sexual risky and offending behavior carried out in groups; see Hart-Kerkhoffs et al. (2011)]. Criminogenic environments (e.g., domestic and community criminogenic exposure) have also been consistently found to be associated with sexual and nonsexual risky and offending behavior (Hewitt and Beauregard, 2014; Chan et al., 2015b).

## The Present Study

Through examining a number of mainstream theoretical principles and concepts, this cross-sectional study explores the psychosocial risk factors that are correlated with general, penetrative, and non-penetrative RSB in Hong Kong male and female young adults. In this study, penetrative RSB is simply referred to sexual behavior that involved sexual penetration (e.g., vaginal sex), while sexual behavior that does not involve sexual penetration is regarded as non-penetrative RSB (e.g., breast fondling). General RSB is the overall sexual behavior that involves both penetrative and non-penetrative RSB. The study makes three important contributions. First, it might be the first empirical work to examine different types of RSB by testing several mainstream criminological theories. Second, it explores the gender differences among a large sample of young male and female adults recruited in Hong Kong. Third, the findings can inform practice in the specific area of preventive measures by identifying significant psychosocial risk factors for different types of RSB. Strategic and timely interventions could help to reduce the propensity of young adults to engage in RSB. Drawing from the literature, the following research hypotheses were put forward.

Hypothesis 1: Young male adults are expected to have higher mean levels than young female adults of all types of RSB (i.e., general, penetrative, and non-penetrative behavior) and of most psychosocial risk factors (i.e., negative temperament, alcohol and drug use, paraphilic interest, and perceived neighborhood disorganization), while young female adults are expected to have higher mean levels than young male adults of self-control and social bonds.
Hypothesis 2: Psychosocial risk factors are expected to be correlated with different types of RSB, even after controlling for the young adults' demographic characteristics (i.e., age, religiosity, and marital status), with low levels of self-control and social bonds, and high levels of negative temperament, use of alcohol and other drugs, paraphilic interest, and perceived neighborhood disorganization are expected to be correlated with all types of RSB.

## Methods

### Participants and Procedure

The participants were aged 18 years and above and were students at eight public (i.e., government funded) and two private universities in Hong Kong. Participants were approached randomly within university compounds (e.g., student cafeterias, reading corners, libraries, and common areas) with no preset time period (i.e., about 2 years). The study was approved by the institutional review board of the author's university. A pen-and-paper questionnaire was administered to the participants upon receiving their informed consent. Their participation in the study was completely voluntary, and they were ensured that their anonymous responses would only be used for research purposes. No monetary incentive was provided. The participants took an average of 25 min to complete the questionnaire. The rate of successful responses for this questionnaire survey was ~90%.

A total of 1,171 valid questionnaires were collected. Of the participants, 58.5% were females and 41.5% were males (see Table 1). The mean age was 20.95 years (*SD* = 2.01, range = 18–40). A slight gender difference was observed, with the mean ages of male and female participants being 21.24 years (*SD* = 2.14) and 20.75 years (*SD* = 1.88), respectively (*t* = 3.98, *p* < 0.001). A large majority of the participants (88.6%) were from Hong Kong, with the remainder either from mainland China (8.5%) or other countries (2.9%; e.g., Canada, Macau, Japan, Australia, and USA). Nearly two-thirds of the participants (62.1%) reported that they were currently single, slightly more than half (57.6%) were post-secondary school educated, and ~3 quarters of them were without any religious affiliation (73.1%).

**Table 1:** Sample demographic characteristics (*N* = 1,171).

**Variable**	***N***	**Percentage**
**Gender**	(*N* = 1160)	
Male	481	41.5%
Female	679	58.5%
**Country of origin**	(*N* = 1154)	
Hong Kong	1023	88.6%
Mainland China	98	8.5%
Others	33	2.9%
(e.g., Canada, Macau, Japan, Australia, and USA)		
**Marital status**	(*N* = 1157)	
Single	718	62.1%
Nonsingle	439	37.9%
**Highest education attainment**	(*N* = 1156)	
Secondary school education	492	42.4%
Post-secondary school education	664	57.6%
(e.g., associate degree/ high diploma; and		
undergraduate and postgraduate degrees)		
**Religious belief**	(*N* = 1154)	
Without a religious belief	844	73.1%
With a religious belief	310	26.9%
(e.g., Christianity, Catholic, Buddhism, Muslim)		

### Measures

Self-reported measures were used to explore (a) the participants' prevalence of general, penetrative, and non-penetrative RSB; (b) the gender differences in these three types of RSB; and (c) the general and gendered effects of psychosocial risk factors in association with general, penetrative, and non-penetrative RSB. The questionnaires were printed in both English and Chinese versions to accommodate the participants' different language abilities. To accommodate the local Chinese population, the English-language measures were first translated by an academically qualified and experienced English-to-Chinese translator. The Chinese versions were then back-translated to English to ensure face validity, and the back-translation was compared with the original English-language scales to determine their content similarity.

#### Risky Sexual Behavior

The Sexual Risk Survey (SRS; Turchik and Garske, 2009) was adopted with slight modifications to assess the participants' levels of involvement in RSB over the past 6 months. Each item on the 23-item measure (with 16 items for penetrative and seven items for non-penetrative RSB) was dichotomized (0 = *no*, 1 = *yes*), with a higher overall score indicating a greater involvement in RSB. A total score ranged from 0 to 23. Sample items asked whether the participants “Had left a social event with someone he/she just met” (non-penetrative RSB), “Had anal sex without a condom,” (penetrative RSB), and “Had sex with someone you don't know well or just met” (penetrative RSB). The Cronbach's α coefficient for the measure was 0.69 (males = 0.72, females = 0.60).

#### Self-Control Scale

The six original elements of self-control (risk-seeking, impulsivity, volatile temper, self-centeredness, preference for physical activities, and preference for simple tasks), as theorized in Gottfredson and Hirschi's (1990) self-control theory, are commonly known as indicators of low self-control. The 23-item Low Self-Control Scale (LSCS; Grasmick et al., 1993), measured on a four-point Likert scale (1 = *strongly agree*, 4 = *strongly disagree*), was adopted to measure the participants' self-control levels. A total score on the LSCS ranges from 23 to 92, with a higher score denoting greater self-control. Sample items were “I always do whatever brings me pleasure here and now, even at the cost of some distant goal,” “When I am really angry, other people better stay away from me,” and “Excitement and adventure are more important to me than security.” The α coefficient of LSCS was 0.87 (males = 0.89, females = 0.85).

#### Negative Temperament Scale

To measure the participants' levels of negative feelings, a scale with eight items assessed on a four-point Likert scale (1 = *strongly agree*, 4 = *strongly disagree*) was used. The total score ranged from 8 to 32, with a higher score indicating a more strongly negative temperament (Tillyer and Wright, 2014). Sample items were “I get stressed out easily,” “I get upset easily,” and “I frequently have mood swings.” The internal consistency of this measure was 0.64 (males = 0.64, females = 0.65).

#### Alcohol and Drug Use Scale

The use of alcohol and other drugs by participants over the past 30 days was measured using eight items. Items on this scale were scored on a six-point scale (0 = *never*, 5 = *20 or more times*), with a total score ranging from 0 to 40 (Espelage et al., 2014). A higher score signified a higher frequency of alcohol and drug use. Sample items were “Drunk wine or wine coolers (more than a sip or taste),” “Used marijuana (like pot, hash, and reefer),” and “Smoked cigarettes.” The Cronbach's α value of this measure was 0.80 (males = 0.80, females = 0.80).

#### Paraphilic Interests Scale

To assess the participants' interest in paraphilic activities, the 40-item Paraphilia Scale was adopted (Dawson et al., 2016). Items on this scale were measured on a seven-point scale (−3 = *very offensive*, +3 = *very arousing*), with a total score ranging from −120 to +120. A higher score indicated a greater interest in paraphilic activities. Sample items were “You are having your feet kissed, fondled, and touched,” “You are spanking, beating, or whipping someone,” and “You are having sex with a boy below the age of 12.” The Cronbach's α value of this measure was 0.98 (males = 0.98, females = 0.98).

#### Social Bonding Scale

Based on Hirschi's (1969) social control theory, Chapple et al. (2005) developed an 18-item Social Bonding Scale (SBC) to assess the participants' conventional ties and attachments to their parents, peers, school, and society in general. The parental attachment component in the SBC was extracted into two separate latent constructs: parental dependence and parental bonding. With a total score ranging from 18 to 38, the SBC items were measured on either a four-point (1 = *never*, 4 = *many times*; two items) or a five-point (1 = *strongly disagree*, 5 = *strongly agree*; 16 items) Likert format. A higher score denoted a greater social bond. Sample items were “I respect my best friends' opinions about the important things in life,” “I talk over future plans with my parents,” and “I have lots of respect for the police.” The α coefficient of this measure was 0.72 (males = 0.72, females = 0.71).

#### Perceptions of Neighborhood Disorganization Scale

To measure the participants' living environment, a five-item scale was used to evaluate their perception of neighborhood disorganization (Posick, 2013). These items were measured on a four-point Likert scale (1 = *strongly agree*, 4 = *strongly disagree*), with a total score ranging from 5 to 20. Sample items were, “There is a lot of fighting,” “There is a lot of graffiti,” and “There is a lot of crime in my neighborhood.” These items were reverse coded so that higher scores would indicate higher levels of perceived neighborhood disorganization. The internal consistency of this scale was 0.93 (males = 0.93, females = 0.92).

### Analytic Strategy

Using SPSS version 27.0, independent sample *t*-tests were first computed to assess the gender differences in different types of RSB (i.e., general, penetrative, and non-penetrative behavior) and psychosocial risk factors (i.e., self-control, negative temperament, use of alcohol and other drugs, paraphilic interest, social bonding, and perceived neighborhood disorganization). Pearson correlations were subsequently computed to examine the links among general, penetrative, and non-penetrative RSB. Finally, ordinary least square (OLS) regressions were performed to investigate the effects of different psychosocial risk factors on general, penetrative, and non-penetrative RSB while controlling for the participants' demographic characteristics (i.e., age, religiosity, and marital status). Religiosity was measured on a six-point Likert format, asking how religious the participants perceived they were (1 = *not at all*, 6 = *very strongly*). The significance level was set at 0.05. Given only nine psychosocial predictor variables tested in this study, the large sample size in this study was found to generate sufficient power. Pearson correlations of the tested constructs were computed, and no correlation at or above 0.70 was observed, indicating no collinearity. Other OLS assumptions (e.g., normality of independent and dependent variables, absence of outliers, homoscedasticity, and residuals' independence) were also tested and found no violation. No data imputation was performed on the missing data as the amount of missing data was small (i.e., <10%) and was regarded as missing at random.

### Ethical Considerations

This study was approved by the ethical committee of the author's university. Participants could end their participation, contact the primary investigator, and/or receive professional counseling at any time. Data were collected anonymously with no personal identifying details recorded.

## Results

### Mean Differences Between Types of RSB and Psychosocial Risk Factors

Table 2 presents the mean scores for the different types of RSB and psychosocial risk factors of male and female participants. Male participants reported significantly higher levels of general (*t* = 2.69, *p* = 0.007), penetrative (*t* = 2.62, *p* = 0.009), and non-penetrative (*t* = 2.24, *p* = 0.025) RSB than female participants. Concerning the psychosocial characteristics, male participants scored significantly higher in negative temperament (*t* = 3.07, *p* = 0.002), use of alcohol and other drugs (*t* = 4.72, *p* < 0.001), and paraphilic interest (*t* = 3.14, *p* = 0.002) than their female counterparts. However, female participants reported higher levels of self-control (*t* = −3.36, *p* < 0.001) and social bonding (*t* = −5.73, *p* < 0.001).

**Table 2:** Gender differences of the prevalence of self-reported risky sexual behavior and psychosocial characteristics.

**Variable**	**All sample**		**Male**		**Female**		***t*-value**
	**(*****N*** **=** **1,171)**		**(*****N*** **=** **481)**		**(*****N*** **=** **679)**		
	***M***	***SD***	***M***	***SD***	***M***	***SD***	
**Risky sexual behavior**							
General behavior	1.34	2.94	1.59	3.29	1.12	2.39	2.69^**^
Penetrative behavior	0.80	1.97	0.96	2.20	0.65	1.58	2.62^**^
Non-penetrative behavior	0.54	1.21	0.63	1.35	0.47	1.05	2.24^*^
**Psychosocial risk factors**							
Self-control	61.26	8.35	60.28	9.16	62.00	7.64	−3.36^***^
Negative temperament	20.01	2.76	20.30	2.75	19.80	2.75	3.07^**^
Alcohol and drug use	2.94	4.18	3.65	4.74	2.44	3.66	4.72^***^
Paraphilic interest	−73.55	41.55	−69.12	42.90	−76.96	40.20	3.14^**^
Social bonding	58.17	7.47	56.72	7.55	59.24	7.23	−5.73^***^
Disorganized neighborhood	8.01	2.78	7.99	2.95	8.03	2.63	−0.23

* *p < 0.05*,

** *p < 0.01*,

*** *p < 0.001*.

### Pearson Correlations of Penetrative and Non-penetrative RSB

Pearson correlations were computed to assess the relationships between penetrative and non-penetrative RSB. The results shown in Table 3 indicate that both subtypes of RSB were significantly and positively correlated with one another, in the range of 0.64 to 0.69.

**Table 3:** Pearson correlations of self-reported risky sexual behavior.

**Type of risky sexual behavior**	**PB**	**NB**
**All sample (*****N*** **=** **1171)**		
Penetrative behavior (PB)	1.00	
Non-penetrative behavior (NB)	0.69^**^	1.00
**Male (*****N*** **=** **481)**		
Penetrative behavior (PB)	1.00	
Non-penetrative behavior (NB)	0.69^**^	1.00
**Female (*****N*** **=** **679)**		
Penetrative behavior (PB)	1.00	
Non-penetrative behavior (NB)	0.64^**^	1.00

** *p < 0.01*.

### Effects of Psychosocial Risk Factors on General, Penetrative, and Non-penetrative RSB

Table 4 presents OLS regressions that were computed to examine the effects of psychosocial characteristics on self-reported general, penetrative, and non-penetrative RSB, while controlling for the participants' demographic characteristics (i.e., age, religiosity, and marital status). All OLS regression models were significant. Overall, the participants' levels of alcohol and drug use (*B* = 0.26, *SE* = 0.02, *p* < 0.001) and paraphilic interest (*B* = 0.01, *SE* = 0.01, *p* < 0.001), and being older (*B* = 0.11, *SE* = 0.04, *p* = 0.002), less religious (*B* = −0.14, *SE* = 0.05, *p* = 0.003), and not single (*B* = −0.69, *SE* = 0.15, *p* < 0.001) were significantly associated with their levels of RSB. Broken down by gender, participants' levels of alcohol and drug use (male: *B* = 0.24, *SE* = 0.03, *p* < 0.001; female: *B* = 0.29, *SE* = 0.02, *p* < 0.001) and paraphilic interest (male: *B* = 0.01, *SE* = 0.01, *p* < 0.001; female: *B* = 0.01, *SE* = 0.01, *p* = 0.039), and not being single (male: *B* = −0.73, *SE* = 0.27, *p* = 0.006; female: *B* = −0.75, *SE* = 0.17, *p* < 0.001) were positively correlated with self-reported RSB for both males and females. Moreover, being older (*B* = 0.18, *SE* = 0.06, *p* = 0.003) and being less religious (*B* = −0.27, *SE* = 0.09, *p* = 0.002) were significantly correlated with male participants' general RSB, while level of perceived neighborhood disorganization (*B* = −0.08, *SE* = 0.03, *p* = 0.019) was negatively associated with female participants' self-reported RSB. A gender difference was thus observed in regard to perceived neighborhood disorganization, which was only a significant predictor of general RSB among female participants.

**Table 4:** OLS regression models of self-reported risky sexual behavior.

**Predictors**	**General behavior**			**Penetrative behavior**			**Non-penetrative behavior**		
	**All sample**	**Male**	**Female**	**All sample**	**Male**	**Female**	**All sample**	**Male**	**Female**
	***B* (*SE*)**	***B* (*SE*)**	***B* (*SE*)**	***B* (*SE*)**	***B* (*SE*)**	***B* (*SE*)**	***B* (*SE*)**	***B* (*SE*)**	***B* (*SE*)**
**Demographic characteristics**									
Age	0.11 (0.04)^**^	0.18 (0.06)^**^	0.06 (0.04)	0.10 (0.02)^***^	0.13 (0.04)^**^	0.07 (0.03)^*^	0.01 (0.02)	0.05 (0.03)	−0.01 (0.02)
Religiosity	−0.14 (0.05)^**^	−0.27 (0.09)^**^	−0.06 (0.06)	−0.12 (0.03)^***^	−0.18 (0.06)^**^	−0.08 (0.04)^*^	−0.02 (0.02)	−0.08 (0.04)^*^	0.02 (0.03)
Marital status	−0.69 (0.15)^***^	−0.73 (0.27)^**^	−0.75 (0.17)^***^	−0.56 (0.10)^***^	−0.61 (0.18)^**^	−0.58 (0.11)^***^	−0.13 (0.07)^*^	−0.13 (0.12)	−0.16 (0.08)^*^
(0 = non-single, 1 = single)									
**Psychosocial risk factors**									
Self-control	−0.01 (0.01)	−0.01 (0.02)	0.01 (0.01)	0.01 (0.01)	0.01 (0.01)	0.01 (0.01)	−0.01 (0.01)	−0.01 (0.01)	−0.01 (0.01)
Negative temperament	−0.05 (0.03)	−0.02 (0.05)	−0.05 (0.03)	−0.04 (0.02)^*^	−0.04 (0.03)	−0.03 (0.02)	−0.01 (0.01)	0.01 (0.02)	−0.02 (0.01)
Alcohol and drug use	0.26 (0.02)^***^	0.24 (0.03)^***^	0.29 (0.02)^***^	0.16 (0.01)^***^	0.15 (0.02)^***^	0.17 (0.02)^***^	0.10 (0.01)^***^	0.09 (0.01)^***^	0.12 (0.01)^***^
Paraphilic interest	0.01 (0.01)^***^	0.01 (0.01)^***^	0.01 (0.01)^*^	0.01 (0.01)^***^	0.01 (0.01)^***^	0.01 (0.01)	0.01 (0.01)^***^	0.01 (0.01)^**^	0.01 (0.01)^*^
Social bonding	−0.02 (0.01)	−0.02 (0.02)	−0.02 (0.01)	−0.01 (0.01)	−0.02 (0.01)	−0.01 (0.01)	−0.01 (0.01)	−0.01 (0.01)	−0.01 (0.01)
Disorganized neighborhood	−0.02 (0.03)	0.04 (0.05)	−0.08 (0.03)^*^	−0.02 (0.02)	0.01 (0.03)	−0.05 (0.02)^*^	−3.96 (0.01)	0.04 (0.02)	−0.03 (0.02)^*^
Adjusted *R*^2^	0.25	0.25	0.27	0.23	0.23	0.24	0.20	0.19	0.22
*F*	43.19^***^	18.00^***^	27.51^***^	38.50^***^	16.16^***^	23.85^***^	31.33^***^	13.09^***^	20.93^***^

*Unstandardized beta (B) and standard error (SE)*.

* *p < 0.05*,

** *p < 0.01*,

*** *p < 0.001*.

Concerning penetrative RSB, the participants' levels of negative temperament (*B* = −0.04, *SE* = 0.02, *p* = 0.043), alcohol and drug use (*B* = 0.16, *SE* = 0.01, *p* < 0.001), and paraphilic interest (*B* = 0.01, *SE* = 0.01, *p* < 0.001), and being older (*B* = 0.10, *SE* = 0.02, *p* < 0.001), less religious (*B* = −0.12, *SE* = 0.03, *p* < 0.001), and not single (*B* = −0.56, *SE* = 0.10, *p* < 0.001) were found to be significant predictors. Broken down by gender, level of alcohol and drug use (male: *B* = 0.15, *SE* = 0.02, *p* < 0.001; female: *B* = 0.17, *SE* = 0.02, *p* < 0.001) and being older (male: *B* = 0.13, *SE* = 0.04, *p* = 0.001; female: *B* = 0.07, *SE* = 0.03, *p* = 0.012), less religious (male: *B* = −0.18, *SE* = 0.06, *p* = 0.002; female: *B* = −0.08, *SE* = 0.04, *p* =0.035), and not single (male: *B* = −0.61, *SE* = 0.18, *p* = 0.001; female: *B* = −0.58, *SE* = 0.11, *p* < 0.001) were positively correlated with penetrative RSB for both males and females. Furthermore, male participants' tendency to engage in penetrative RSB was also positively associated with their level of paraphilic interest (*B* = 0.01, *SE* = 0.01, *p* < 0.001), while the level of perceived neighborhood disorganization (*B* = −0.05, *SE* = 0.02, *p* = 0.033) was negatively correlated with penetrative RSB in female participants. Gender differences were thus found in paraphilic behavior, which was significantly associated with penetrative RSB only in male participants, and in perceived neighborhood disorganization, which was only a significant predictor in female participants.

Several significant predictors were found for the participants' non-penetrative RSB. The participants' levels of alcohol and drug use (*B* = 0.10, *SE* = 0.01, *p* < 0.001) and paraphilic interest (*B* = 0.01, *SE* = 0.01, *p* < 0.001), and not being single (*B* = −0.13, *SE* = 0.07, *p* = 0.048) were significantly associated with their involvement in non-penetrative RSB. For male participants, levels of alcohol and drug use (*B* = 0.09, *SE* = 0.01, *p* < 0.001) and paraphilic interest (*B* = 0.01, *SE* = 0.01, *p* = 0.001), and being less religious (*B* = −0.08, *SE* = 0.04, *p* = 0.028) were positively correlated with their propensity to engage in non-penetrative RSB. For female participants, levels of alcohol and drug use (*B* = 0.12, *SE* = 0.01, *p* < 0.001), paraphilic interest (*B* = 0.01, *SE* = 0.01, *p* = 0.038), and perceived neighborhood disorganization (*B* = −0.03, *SE* = 0.02, *p* = 0.046), and not being single (*B* = −0.16, *SE* = 0.08, *p* = 0.035) were significantly associated with the extent of their non-penetrative RSB. A gender difference was thus found with perceived neighborhood disorganization only significantly associated with non-penetrative RSB among female participants.

## Discussion

This study has offered an initial insight into the extent of RSB in a Hong Kong sample. It is important not only for its contribution to the body of knowledge on the topic, but also and more specifically for its focus on the under-researched population of Hong Kong young adults. There were two purposes of the study: (1) to explore gender differences at the mean levels in different types of RSB (i.e., general, penetrative, and non-penetrative) and psychosocial risk factors (i.e., self-control, negative temperament, the use of alcohol and other drugs, paraphilic interest, social bonds, and perceived neighborhood disorganization), and (2) to examine whether the relationships between different types of RSB and psychosocial risk factors hold when controlling for demographic characteristics (i.e., age, religiosity, and marital status). In general, male participants reported significantly more general, penetrative, and non-penetrative RSB than female participants. Nevertheless, their mean scores were relatively low. Compared to female participants, male participants had significantly higher levels of negative temperament, alcohol and drug use, and paraphilic interest but lower levels of self-control and social bonds. In other words, male participants were found to possess a higher probability than female participants to engage in RSB.

In addition, there were several noteworthy findings with respect to the role of psychosocial risk factors on the propensity to engage in RSB. Thus, the findings lend support to some major criminological theoretical propositions in explaining RSB. It is, however, should be noted that some theoretical models—i.e., self-control and social control theories—failed to find support in this study. More research is required as to whether it is merely due to the sampling strategy or the inherent lack of comprehensiveness of the theoretical framework to explain RSB. To a large extent, the male and female participants in this study shared a similar set of psychosocial risk factors for their involvement in general, penetrative, and non-penetrative RSB. The participants' use of alcohol and drugs and paraphilic interests were generally useful in explaining all types of RSB (with the exception of female participants' tendencies to engage in penetrative RSB). Put differently, using alcohol and other drugs and having paraphilic interests were found to be strong predictors of an individual's likelihood to engage in all categories of RSB. According to the routine activity and lifestyle approach, individuals are more likely to have a heightened risk of engaging in RSB if they spend substantial time in “unstructured socializing” (e.g., drinking alcohol and taking drugs) with their deviant peers (Schreck et al., 2004). This finding is also consistent with the learning approach, in which RSB is said to be learned through differential association and behavioral imitation (Akers, 1997; Warr, 2002). In the present study, the association between the use of alcohol and other drugs and RSB was particularly strong among male participants. Masculine identity is often strengthened by engaging in risky and deviant behavior (Messerschmidt, 1993). Cultural influences on males may encourage competitive pursuits that are often risky and deviant in nature, and male group membership typically emphasizes risk-taking, toughness, aggression, and physical strength (Augustyn and McGloin, 2013).

Moreover, an individual's sexual risk-taking tendency was found to be positively associated with his/her development of paraphilic interests. Previous studies have found that individuals who had paraphilic interests or had been diagnosed with paraphilias were more likely to engage in hypersexual activities, such as having multiple sexual partners, a high sexual appetite and preoccupation, and high rates of sexual behavior (Kafka and Hennen, 2003; Långström and Hanson, 2006; Kafka, 2009; Chan, 2021). Indeed, Chan (2020a) found that high levels of RSB and paraphilic interests were significantly associated with sexual offending behavior. Relative to previous studies, participants in this study reported comparatively lower level of paraphilic interests. Interestingly, female participants' paraphilic interests failed to find support for their specific involvement in penetrative RSB, although this psychosocial risk factor has demonstrated significance in general and non-penetrative RSB. Perhaps the cultural and societal norms have played a significant impact on the recognition and acceptance of behavior as normal or deviance. Asian and Middle Eastern cultures commonly adopt a higher restrictive view on sexual issues, where discussion on sex has always been and remains a taboo subject (Ho et al., 2011; Baazeem, 2016). This is particularly relevant to females in these cultures where they are expected to live up to the cultural gender role in view of the sexual conservatism within their cultural background. It is reasonable to posit that penetrative RSB may seem to be more culturally sensitive than non-penetrative behavior.

Negative temperament was found to be a significant risk factor of penetrative RSB. In other words, those who often experienced negative emotions had a higher likelihood of engaging in penetrative RSB (e.g., unprotected sex, sex with multiple partners). This finding is consistent with the literature. Studies have demonstrated that individuals who exhibited high levels of emotion regulation deficit or negative affect (e.g., neuroticism, depression, anxiety, shame, and guilt) were more likely to adopt maladaptive behavior, such as RSB, in coping with their negative emotions (Leith and Baumeister, 1996; Auerbach et al., 2007; Stuewig et al., 2015). Similarly, a positive relationship has been identified between negative emotions and the perpetration of delinquent and criminal behavior (Chui and Chan, 2012; DeLisi and Vaughn, 2014; Wolff et al., 2016), particularly in sexual offending behavior (e.g., violent sexual offenses, sexual homicides; Chan et al., 2015a; Chan and Beauregard, 2016; Chan, 2020b). Indeed, the perpetration of (penetrative) RSB has been found to be a significant predictor of subsequent sexual offending behavior (Lussier and Cale, 2013; Smallbone and Cale, 2015). Nonetheless, this finding should be studied cautiously especially when no sex difference was found. It is possible that negative temperament reached significance due to an increased sample size in the full sample.

Interestingly, perceived neighborhood disorganization was only significantly associated with RSB among female participants. Put differently, female participants who perceived their neighborhood to be disorganized (e.g., that they were living in a crime-prone environment) were more likely to engage in RSB. Previous research on deviant and criminal behavior indicates that inherently maladaptive lifestyles are likely to contribute to forming a platform for learning maladaptive behaviors [e.g., RSB and the use of alcohol and other drugs; Hoeben et al., 2016; see Chan (2019b)]. Mills (2003) asserted that “people who are exposed to violence (or criminogenic environment) are more likely to absorb pro-violence (or pro-deviance) norms and values, which, in turn, makes them more violence (or deviant) prone” (p. 88). A similar finding was also noted in Chan's (2019c) study of young people whereby pro-violence attitudes, deviant peer influence, and alcohol and drug use were significantly associated with violent, nonviolent, and general delinquent behavior. Relative to the male participants, perhaps the female participants in this study might have been exposed to more risky and deviant incidents (e.g., deviant peer association with a high prevalence of RSB) recently or in the past and that this has shaped their perception of residing in a disorganized neighborhood. Thus, this perception predicts their engagement in RSB.

Caution should be exercised in interpreting the findings of this study in view of several limitations. First, this was a cross-sectional study, and hence, the findings can only be interpreted in correlational terms. Future studies could consider adopting a longitudinal framework to better understand the causal relationships between the participants' psychosocial risk factors and their self-reported RSB. Besides, it is possible that there are other potential confounders of the associations between RSB and significant psychosocial risk factors. Future research could also examine the underlying mechanism of the target associations, such as the potentially mediating role of variables included in regression models of this study or other theoretically relevant variables. Second, this study was limited by the use of self-reported data. Social desirability and memory recall biases may lead to underreporting of RSB and other sexual interests (e.g., paraphilic interests). Thus, a measure of response bias could be considered in future studies to minimize the potential effects of reporting biases. Other potential limitations in data collection may include the possibility of underreporting, possible differences in underreporting between the sexes, differences between those who agreed to complete the questionnaire and those that did not, and issues of anonymity (e.g., level of anonymity and perceived anonymity). Moreover, sexual orientation of the participants was not controlled for in the analysis, which was found to have a substantial association between non-heterosexual orientation and engaging in RSB in previous studies (e.g., Leigh, 1990). Hence, future research could consider exploring its potential effects on RSB. Finally, the sample comprised only university students. The findings are not necessarily generalizable to the wider Hong Kong population or even to the young adult population. However, the sample can perhaps be regarded as representative of the wider population of Hong Kong university students given that the sample was recruited from all universities in Hong Kong.

### Implications of the Findings

The adverse consequences of RSB should not be overlooked, and the findings of this study have important implications for practice. The public awareness of STIs, such as HIV, should be enhanced, particularly among late adolescents and young adults but also targeted much earlier in life. The school-based sexuality education in Hong Kong has long been criticized for its lack of comprehensiveness to promote an effective sexual education (Andres et al., 2021). Therefore, it is even more important that school-based prevention programs for school pupils and university students should include educational materials on the risks associated with the premature onset of sexual activities and the use of alcohol and other drugs (Staton et al., 1999). The need for specific behavioral changes, such as decreasing the number of sexual partners, decreasing alcohol consumption and drug use before sex, negotiating condom use for safer sex, and transforming attitudes about safer and more socially acceptable sexual practices (i.e., nonparaphilic activities), is a critical educational message to deliver to the younger population. Issues relevant to hypersexuality and atypical sexual interests (i.e., paraphilic interests) should also be addressed to help young people to better understand the important role of other aspects of sexuality, such as general sexual inhibition and excitation, in the development of paraphilic interests (Dawson et al., 2016). Resources for positive and prosocial self-development and counseling services should be strengthened to encourage those who need to deal with negative emotions to do so in an effective and timely manner.

Community-based preventive efforts in the same directions are also needed to target young people who are not enrolled in university or have dropped out of school (i.e., at-risk youth). Youth and social workers in the community should strive to convey these messages using available resources, such as regular community activities and youth programs (Chan and Chui, 2012, 2015). Public seminars regarding psychological and emotional health should be conducted regularly to disseminate useful information to effectively cope with negative emotions (e.g., anger management, self-assertiveness, and general positive emotions). For individuals who are at a high risk of or are currently engaging in RSB, social norm interventions should be conducted to address misperceptions over alcohol consumption, drug use, RSB (Martens et al., 2006), and paraphilic activities (Chan, 2021). Ideally, this intervention may not only have a remedial effect by reducing the frequency with which individuals who already engage in a behavior choose to do so (e.g., RSB, use of alcohol and other drugs, paraphilic activities), but also have a preventive effect by correcting misperceptions among those who are not yet frequently engaging in such behavior. Raising their awareness and skills may also help to deter them from potential domestic (familial) and community (neighborhood) criminogenic influences.

## Data Availability Statement

The raw data supporting the conclusions of this article will be made available by the authors, without undue reservation.

## Ethics Statement

The studies involving human participants were reviewed and approved by Human Subjects Ethics Sub-Committee, City University of Hong Kong. The patients/participants provided their written informed consent to participate in this study.

## Author Contributions

The author confirms being the sole contributor of this work and has approved it for publication.

## Conflict of Interest

The author declares that the research was conducted in the absence of any commercial or financial relationships that could be construed as a potential conflict of interest.

## References

[B1] Abajobir A. A. Kisely S. Maravilla J. C. Williams G. Najman J. M. (2017). Gender differences in the association between childhood sexual abuse and risky sexual behaviours: a systematic review and meta-analysis. Child Abuse Neglect 63, 249–260. 10.1016/j.chiabu.2016.11.023 27908449

[B2] Agnew R. (1992). Foundation for a general strain theory of crime and delinquency. Criminology 30, 47–88. 10.1111/j.1745-9125.1992.tb01093.x

[B3] Agnew R. (2002). Experienced, vicarious, and anticipated strain: an exploratory study on physical victimization and delinquency. Justice Q. 19, 603–632. 10.1080/07418820200095371

[B4] Agnew R. Brezina T Wright J. P Cullen F. T. (2002). Strain, personality traits, and delinquency: Extending general strain theory. Criminology 40, 43–72. 10.1111/j.1745-9125.2002.tb00949.x

[B5] Akers R. L. (1985). Deviant Behavior: A Social Learning Approach. Belmont, CA: Wadsworth Publishing.

[B6] Akers R. L. (1997). Criminological Theories: Introduction and Evaluation, 2nd Edn. Los Angeles, CA: Roxbury.

[B7] Akers R. L. (1998). Social Learning and Social Structure: A General Theory of Crime and Deviance. Boston, MA: Northeastern University Press.

[B8] Andres E. B. Choi E. P. H. Fung A. W. C. Lau K. W. C. Ng N. H. T. Yeung M.. (2021). Comprehensive sexuality education in Hong Kong: study protocol for process and outcome evaluation. BMC Public Health 21:197. 10.1186/s12889-021-10253-6 33482802 PMC7820515

[B9] Auerbach R. P. Abela J. R. Z. Ho M. R. (2007). Responding to symptoms of depression and anxiety: emotion regulation, neuroticism, and engagement in risky behaviors. Behav. Res. Therapy 45, 2182–2191. 10.1016/j.brat.2006.11.002 17181999

[B10] Auerbach R. P. Claro A. Abela J. R. Zhu X. Yao S. (2010). Understanding risky behavior engagement amongst Chinese adolescents. Cogn. Therapy Res. 34, 159–167. 10.1007/s10608-009-9238-x

[B11] Augustyn M. B. McGloin J. M. (2013). The risk of informal socializing with peers: considering gender differences across predatory delinquency and substance use. Justice Quart. 30, 117–143. 10.1080/07418825.2011.597417

[B12] Baazeem A. (2016). Challenges to practicing sexual medicine in the Middle East. Sexual Med. Rev. 4, 221–228. 10.1016/j.sxmr.2016.04.001 27871955

[B13] Bandura A. (1973). Aggression: A Social Learning Analysis. Englewood Cliffs, NJ: Prentice Hall.

[B14] Chan H. C. O. (2015). Understanding Sexual Homicide Offenders: An Integrated Approach. Basingstoke: Palgrave Macmillan. 10.1057/9781137453723

[B15] Chan H. C. O. (2019a). A Global Casebook of Sexual Homicide. Singapore: Springer Nature. 10.1007/978-981-13-8859-0

[B16] Chan H. C. O. (2019b). Exploring the overlap between victimization and offending among Hong Kong adolescents. J. Criminal Justice 61, 72–80. 10.1016/j.jcrimjus.2019.03.003

[B17] Chan H. C. O. (2019c). Violent offending, nonviolent offending, and general delinquency: exploring the criminogenic risk factors of Hong Kong male and female adolescents. Int. J. Offender Therapy Comparative Criminol. 10.1177/0306624X19881917. [Epub ahead of print]. 31602999

[B18] Chan H. C. O. (2020a). The victim-offender overlap in sexual offending: exploring a community-based sample of young adults in Hong Kong. Sexual Abuse. 10.1177/1079063220981889. [Epub ahead of print]. 33353485

[B19] Chan H. C. O. (2020b). Sexual sadism and psychopathy in sexual homicide, in The Wiley International Handbook of Psychopathic Disorders and the Law, 2nd Edn, eds A. Felthous and H. Sass (Hoboken, NJ: John Wiley & Sons), 693–712. 10.1002/9781119159322.ch30

[B20] Chan H. C. O. (2021). Paraphilic interests: the role of psychosocial factors in a sample of young adults in Hong Kong. Sexuality Res. Soc. Policy. 10.1007/s13178-020-00532-z. [Epub ahead of print].

[B21] Chan H. C. O. Beauregard E. (2016). Nonhomicidal and homicidal sexual offenders: prevalence of maladaptive personality traits and paraphilic behaviors. J. Interpersonal Violence 31, 2259–2290. 10.1177/0886260515575606 25818862

[B22] Chan H. C. O. Beauregard E. Myers W. C. (2015a). Single-victim and serial sexual homicide offenders: differences in crime, paraphilias, and personality traits. Criminal Behav. Mental Health 25, 66–78. 10.1002/cbm.1925 25111158

[B23] Chan H. C. O. Chui W. H. (2012). Psychological correlates of violent and nonviolent Hong Kong juvenile probationers. Behav. Sci. Law 30, 103–120. 10.1002/bsl.2003 22362616

[B24] Chan H. C. O. Chui W. H. (2013). Social bonds and school bullying: a study of Macanese male adolescents on bullying perpetration and peer victimization. Child Youth Care Forum 42, 599–616. 10.1007/s10566-013-9221-2

[B25] Chan H. C. O. Chui W. H. (2015). Social bonds and self-reported nonviolent and violent delinquency: a study of traditional low risk, at-risk and adjudicated male Chinese adolescents. Child Youth Care Forum 44, 711–730. 10.1007/s10566-015-9303-4

[B26] Chan H. C. O. Chui W. H. (2017). The influence of low self-control on violent and nonviolent delinquencies: a study of male adolescents from two Chinese societies. J. Forensic Psychiatry Psychol. 28, 599–619. 10.1080/14789949.2015.1012534

[B27] Chan H. C. O. Heide K. M. Beauregard E. (2011). What propels sexual murderers: a proposed integrated theory of social learning and routine activities theories. Int. J. Offender Therapy Comparative Criminol. 55, 228–250. 10.1177/0306624X10361317 20160008

[B28] Chan H. C. O. Lo T. W. Zhong L. Y. Chui W. H. (2015b). Criminal recidivism of incarcerated male nonviolent offenders in Hong Kong. Int. J. Offender Therapy Comparative Criminol. 59, 121–142. 10.1177/0306624X13502965 24052598

[B29] Chapple C. L. McQuillan J. A. Berdahl T. A. (2005). Gender, social bonds, and delinquency: a comparison of boys' and girls' models. Soc. Sci. Res. 34, 357–383. 10.1016/j.ssresearch.2004.04.003

[B30] Chui W. H. Chan H. C. O. (2012). Criminal recidivism among Hong Kong male juvenile probationers. J. Child Family Stud. 21, 857–868. 10.1007/s10826-011-9546-0

[B31] Cohen L. E. Felson M. (1979). Social change and crime rate trends: a routine activities approach. Am. Sociol. Rev. 44, 588–608. 10.2307/2094589

[B32] Dawson S. J. Bannerman B. A. Lalumière M. L. (2016). Paraphilic interests: an examination of sex differences in a nonclinical sample. Sexual Abuse 28, 20–45. 10.1177/1079063214525645 24633420

[B33] DeLisi M. Vaughn M. G. (2014). Foundation for a temperament-based theory of antisocial behavior and criminal justice system involvement. J. Criminal Justice 42, 10–25. 10.1016/j.jcrimjus.2013.11.001

[B34] DiClemente R. Milhausen R. R. Salazar L. F. Spitalnick J. Sales J. M. Crosby R. A.. (2010). Development of the sexual sensation-seeking scale for African American adolescent women. Int. J. Sexual Health 22, 248–261. 10.1080/19317611.2010.491388

[B35] Ellwanger S. J. Pratt T. C. (2014). Self-control, negative affect, and young driver aggression: an assessment of competing theoretical claims. Int. J. Offender Therapy Comparative Criminol. 58, 85–106. 10.1177/0306624X12462830 23109495

[B36] Espelage D. L. Low S. Rao M. A. Hong J. S. Little T. D. (2014). Family violence, bullying, fighting, and substance use among adolescents: a longitudinal mediational model. J. Res. Adoles. 24, 337–349. 10.1111/jora.12060

[B37] Felson M. Cohen L. (1980). Human ecology and crime: a routine activities approach. Hum. Ecol. 8, 389–406. 10.1007/BF01561001

[B38] Ferreira A. I. Martinez L. F. Guisande A. (2009). Risky behavior, personality traits and road accidents among university students. Eur. J. Educ. Psychol. 2, 79–98. 10.30552/ejep.v2i2.2327690191

[B39] Fetene N. Mekonnen W. (2018). The prevalence of risky sexual behaviors among youth center reproductive health clinics users and non-users in Addis Ababa, Ethiopia: a comparative cross-sectional study. PLoS ONE 13:e0198657. 10.1371/journal.pone.0198657 29879164 PMC5991709

[B40] Freeman N. Muraven M. (2010). Self-control depletion leads to increased risk taking. Soc. Psychol. Personal. Sci. 1, 175–181. 10.1177/1948550609360421

[B41] Gottfredson M. R. Hirschi T. (1990). A General Theory of Crime. Standford, CA: Standford University Press.

[B42] Grasmick H. G. Tittle C. R. Bursik R. J. Arneklev B. J. (1993). Testing the core implications of Gottfredson and Hirschi's general theory of crime. J. Res. Crime Delinquency 30, 5–29. 10.1177/0022427893030001002

[B43] Griffin K. W. Scheier L. M. Acevedo B. Grenard J. L. Botvin G. J. (2012). Long-term effects of self-control on alcohol use and sexual behavior among urban minority young women. Int. J. Environ. Res. Public Health 9, 1–23. 10.3390/ijerph9010001 22470274 PMC3315087

[B44] Hart-Kerkhoffs L. A. Vermeirenm R. R. J. M. Jansen L. M. C. Doreleijers T. A. H. (2011). Juvenile group sex offenders: a comparison of group leaders and followers. J. Interpersonal Violence 26, 3–20. 10.1177/0886260510362882 20442449

[B45] Hewitt A. Beauregard E. (2014). Sexual crime and place: the impact of the environmental context on sexual assault outcomes. J. Criminal Justice 42, 375–383. 10.1016/j.jcrimjus.2014.05.003

[B46] Hirschi T. (1969). Causes of Delinquency. Berkeley, CA: University of California Press.

[B47] Hirschi T. Gottfredson M. (1994). The Generality of Deviance. New Brunswick, NJ: Transaction.

[B48] Ho C. C. Singam P. Hong G. E. Zainuddin Z. M. (2011). Male sexual dysfunction in Asia. Asian J. Androl. 13, 537–542. 10.1038/aja.2010.135 21643001 PMC3739628

[B49] Hoeben E. M. Meldrum R. C. Walker D. Young J. T. (2016). The role of peer delinquency and unstructured socializing in explaining delinquency and substance use: a state-of-the-art review. J. Criminal Justice 47, 108–122. 10.1016/j.jcrimjus.2016.08.001

[B50] Hoyle R. H. Fejfar M. C. Miller J. D. (2000). Personality and sexual risk taking: aquantitative review. J. Personal. 68, 1203–1231. 10.1111/1467-6494.00132 11130738

[B51] Kafka M. P. (1997). A monoamine hypothesis for the pathophysiology of paraphilic disorders. Archiv. Sexual Behav. 26, 343–358. 10.1023/A:1024535201089 9251834

[B52] Kafka M. P. (2001). The paraphilia-related disorders: a proposal for a unified classification of nonparaphilic hypersexuality disorders. Sexual Addict. Compulsivity 8, 227–239. 10.1080/107201601753459937

[B53] Kafka M. P. (2009). Hypersexual disorder: a proposed diagnosis for DSM-V. Archiv. Sexual Behav. 39, 377–400. 10.1007/s10508-009-9574-7 19937105

[B54] Kafka M. P. Hennen J. (2003). Hypersexual desire in males: are males with paraphilias different from males with paraphilia-related disorders? Sexual Abuse 15, 307–321. 10.1177/107906320301500407 14571536

[B55] Kahn R. E. Holmes C. Farley J. P. Kim-Spoon J. (2015). Delay discounting mediates parent-adolescent relationship quality and risky sexual behavior for low self-control adolescents. J. Youth Adoles. 44, 1675–1687. 10.1007/s10964-015-0332-y 26202153 PMC4530086

[B56] Kim H. M. Miller L. C. (2020). Are insecure attachment styles related to risky sexual behavior? A meta-analysis. Health Psychol. 39, 46–57. 10.1037/hea0000821 31724425

[B57] King K. M. Fleming C. B. Monahan K. C. Catalano R. F. (2011). Changes in self-control problems and attention problems during middle school predict alcohol, tobacco, and marijuana use during high school. Psychol. Addict. Behav. 25, 69–79. 10.1037/a0021958 21219040 PMC3074244

[B58] Långström N. Hanson K. R. (2006). High rates of sexual behavior in the general population: correlates and predictors. Archiv. Sexual Behav. 35, 37–52. 10.1007/s10508-006-8993-y 16502152

[B59] Långström N. Seto M. C. (2006). Exhibitionistic and voyeuristic behavior in a Swedish national population survey. Archiv. Sexual Behav. 35, 427–435. 10.1007/s10508-006-9042-6 16900414

[B60] Lalumière M. L. Harris G. T. Quinsey V. L. Rice M. E. (2005). The Causes of Rape: Understanding Individual Differences in the Male Propensity for Sexual Aggression. Washington, DC: American Psychological Association. 10.1037/10961-000

[B61] Lansford J. E. Dodge K. A. Fontaine R. G. Bates J. E. Pettit G. S. (2014). Peer rejection, affiliation with deviant peers, delinquency, and risky sexual behavior. J. Youth Adoles. 43, 1742–1751. 10.1007/s10964-014-0175-y 25150986 PMC4163526

[B62] Laundra K. H. Kiger G. Bahr S. J. (2002). A social development model of serious delinquency: examining gender differences. J. Primary Prev. 22, 389–407. 10.1023/A:1015279607215

[B63] Leigh B. C. (1990). The relationship of substance use during sex to high-risk sexual behavior. J. Sex Res. 27, 199–213. 10.1080/00224499009551552

[B64] Leith K. P. Baumeister R. F. (1996). Why do bad moods increase self-defeating behavior? Emotion, risk taking, and self-regulation. J. Personal. Soc. Psychol. 71, 1250–1267. 10.1037/0022-3514.71.6.1250 8979390

[B65] Lu Y. Yu Y. Ren L. Marshall I. H. (2013). Exploring the utility of self-control theory for risky behavior and minor delinquency among Chinese adolescents. J. Contemporary Criminal Justice 29, 32–52. 10.1177/1043986212471240

[B66] Lussier P. Cale J. (2013). Beyond sexual recidivism: a review of the sexual criminal career parameters of adult sex offenders. Aggression Violent Behav. 18, 445–457. 10.1016/j.avb.2013.06.005

[B67] Marshall W. L. (2007). Diagnostic issues, multiple paraphilias, and comorbid disorders in sexual offenders: their incidence and treatment. Aggression Violent Behav. 12, 16–35. 10.1016/j.avb.2006.03.001

[B68] Martens M. P. Page J. C. Mowry E. S. Damann K. M. Taylor K. K. Cimini M. D. (2006). Differences between actual and perceived student norms: an examination of alcohol use, drug use, and sexual behavior. J. Am. College Health 54, 295–300. 10.3200/JACH.54.5.295-300 16539222

[B69] Martinez G. Copen C. E. Abma J. C. (2011). Teenagers in the United States: Sexual activity, contraceptive use, and childbearing, 2006–2010 National Survey of Family Growth. Vital and Health Statistics (Series 23, Number 31). Available online at: https://www.cdc.gov/nchs/data/series/sr_23/sr23_031.pdf (accessed May 14, 2020). 22256688

[B70] Messerschmidt J. W. (1993). Masculinities and Crime: Critique and Reconceptualization of Theory. Lanham, MD: Rowman and Littlefield.

[B71] Mills L. G. (2003). Insult to Injury: Rethinking Our Responses to Intimate Abuse. Princeton, NJ: Princeton University Press.

[B72] Mokdad A. H. Forouzanfar M. H. Daoud F. Mokdad A. A. Bcheraoui C. E. Moradi-Lakeh M.. (2016). Global burden of diseases, injuries, and risk factors for young people's health during 1990–2013: a systematic analysis for the Global Burden of Disease Study 2013. Lancet 387, 2383–2401. 10.1016/S0140-6736(16)00648-6 27174305

[B73] Muraven M. Pogarsky G. Shmueli D. (2006). Self-control depletion and the general theory of crime. J. Quantitative Criminol. 22, 263–277. 10.1007/s10940-006-9011-1

[B74] Osgood D. W. Wilson J. K. O'Malley P. M. Bachman J. G. Johnston L. D. (1996). Routine activities and individual deviant behavior. Am. Sociol. Rev. 61, 635–655. 10.2307/2096397

[B75] Peterson C. H. Buser T. J. Westburg N. G. (2010). Effects of familial attachment, social support, involvement, and self-esteem on youth substance use and sexual risk taking. Family J. 18, 369–376. 10.1177/1066480710380546

[B76] Pinyopornpanish K. Thanamee S. Jiraporncharoen W. Thaikla K. McDonald J. Aramrattana A.. (2017). Sexual health, risky sexual behavior and condom use among adolescents, young adults, and older adults in Chiang Mai, Thailand: findings from a population based survey. BMC Res. Notes 10:682. 10.1186/s13104-017-3055-1 29202883 PMC5715516

[B77] Posick C. (2013). The overlap between offending and victimization among adolescents: results from the second International Self-Report Delinquency Study. J. Contemporary Criminal Justice 29, 106–124. 10.1177/1043986212471250

[B78] Roth A. M. Armenta R. A. Wagner K. D. Roesch S. C. Bluthenthal R. N. Cuevas-Mota J.. (2015). Patterns of drug use, risky behavior, and health status among persons who inject drugs living in San Diego, California: a latent class analysis. Substance Use Misuse 50, 205–214. 10.3109/10826084.2014.962661 25313832 PMC4356115

[B79] Sanchagrin K. Heimer K. Paik A. (2017). Adolescent delinquency, drinking, and smoking: does the gender of friends matter? Youth Soc. 49, 805–826. 10.1177/0044118X14563050

[B80] Schreck C. J. Fisher B. S. Miller J. M. (2004). The social context of violent victimization: a study of delinquent peer effect. Justice Quarterly 21, 23–48. 10.1080/07418820400095731

[B81] Simons L. G. Sutton T. E. Simons R. L. Gibbons F. X. Murry V. M. (2016). Mechanisms that link parenting practices to adolescents' risky sexual behavior: a test of six competing theories. J. Youth Adoles. 45, 255–270. 10.1007/s10964-015-0409-7 26718543

[B82] Smallbone S. Cale J. (2015). An integrated life-course developmental theory of sexual offending, in Sex offenders: A criminal career approach, eds A. Blockland and P. Lussier (West Sussex: John Wiley & Sons), 43–69. 10.1002/9781118314630.ch3

[B83] Staton M. Leukefeld C. Logan T. Zimmerman R. Lynam D. Milich R.. (1999). Risky sex behaviour and substance use among young adults. Health Social Work 24, 147–154. 10.1093/hsw/24.2.147 10340165

[B84] Stuewig J. Tangney J. P. (2007). Shame and guilt in antisocial and risky behaviors, in The self-conscious emotions: Theory and research, eds J. L. Tracy, R. W. Robins, and J. P. Tangney (New York, NY: The Guilford Press), 371–388.

[B85] Stuewig J. Tangney J. P. Kendall S. Folk J. B. Meyer C. R. Dearing R. L. (2015). Children's proneness to shame and guilt predict risky and illegal behaviors in young adulthood. Child Psychiatry Hum. Dev. 46, 217–227. 10.1007/s10578-014-0467-1 24842762 PMC4239200

[B86] Sutherland E. H. (1947). Principles of Criminology, 4th Edn. Philadelphia, PA: Lippincott.

[B87] Taylor T. J. Peterson D. Esbensen F. A. Freng A. (2007). Gang membership as a risk factor for adolescent violent victimization. J. Res. Crime Delinquency 44, 351–380. 10.1177/002242780730584526769575

[B88] Taylor-Seehafer M. Rew L. (2000). Risky sexual behaviour among adolescent women. J. Specialists Pediatric Nurs. 5, 15–25. 10.1111/j.1744-6155.2000.tb00082.x 10743602

[B89] Tillyer M. S. Wright E. M. (2014). Intimate partner violence and the victim-offender overlap. J. Res. Crime Delinquency 51, 29–55. 10.1177/0022427813484315

[B90] Turchik J. A. Garske J. P. (2009). Measurement of sexual risk taking among college students. Archiv. Sexual Behav. 38, 936–948. 10.1007/s10508-008-9388-z 18563548

[B91] Vazsonyi A. T. Klanjšek R. (2008). A test of self-control theory across different socioeconomic strata. Justice Q. 25, 101–131. 10.1080/07418820801954571

[B92] Vazsonyi A. T. Trejos-Castillo E. Huang L. (2006). Risky sexual behaviors, alcohol use, and drug use: a comparison of eastern and western European adolescents. J. Adoles. Health 39, 753.e1–753.e11. 10.1016/j.jadohealth.2006.05.008 17046515

[B93] Warr M. (2002). Companions in Crime: The Social Aspects of Criminal Conduct. Cambridge, MA: Cambridge University Press. 10.1017/CBO9780511803956

[B94] Windle M. (1994). Substance use, risky behaviors, and victimization among a US national adolescent sample. Addiction 89, 175–182. 10.1111/j.1360-0443.1994.tb00876.x 8173483

[B95] Wolff K. T. Baglivio M. T. Piquero A. R. Vaughn M. G. DeLisi M. (2016). The triple crown of antisocial behavior: effortful control, negative emotionality, and community disadvantage. Youth Violence Juvenile Justice 14, 350–366. 10.1177/1541204015599042

[B96] Wong W. C. W. Zhao Y. Wong N. S. Parish W. L. Miu H. Y. H. Yang L.. (2017). Prevalence and risk factors of chlamydia infection in Hong Kong: a population-based geospatial household survey and testing. PLoS ONE 12:e0172561. 10.1371/journal.pone.0172561 28225805 PMC5321413

[B97] World Health Organization (2019). Sexually transmitted infections (STIs). Available online at: https://www.who.int/en/news-room/fact-sheets/detail/sexually-transmitted-infections-(stis) (accessed May 14, 2020).

[B98] Zapolski T. C. B. Cyders M. A. Smith G. T. (2009). Positive urgency predicts illegal drug use and risky sexual behavior. Psychol. Addict. Behav. 23, 348–354. 10.1037/a0014684 19586152 PMC2709762

